# Unusual striped dolphin mass mortality episode related to cetacean morbillivirus in the Spanish Mediterranean sea

**DOI:** 10.1186/1746-6148-9-106

**Published:** 2013-05-23

**Authors:** Consuelo Rubio-Guerri, Mar Melero, Fernando Esperón, Edwige Nina Bellière, Manuel Arbelo, Jose Luis Crespo, Eva Sierra, Daniel García-Párraga, Jose Manuel Sánchez-Vizcaíno

**Affiliations:** 1 VISAVET Center and Animal Health Department, Veterinary School, Complutense University of Madrid, Av Puerta del Hierro s/n, Madrid 28040, Spain; 2 Animal Health Research Centre, Ctra. de Algete a El Casar s/n, Madrid 28130, Spain; 3 Unit of Histology and Veterinary Pathology, Institute for Animal Health, Veterinary School, University of Las Palmas de Gran Canaria, Carretera de Trasmontaña s/n, Arucas (Las Palmas), Canary Islands 35413, Spain; 4 Veterinary Services, Oceanographic Aquarium of the Ciudad de las Artes y las Ciencias, C/ Junta de murs i valls s/n, Valencia 46023, Spain

**Keywords:** Cetacean morbillivirus, Dolphin morbillivirus, Mediterranean sea, Mass mortality, Striped dolphin

## Abstract

**Background:**

In the last 20 years, Cetacean Morbillivirus (CeMV) has been responsible for many die-offs in marine mammals worldwide, as clearly exemplified by the two dolphin morbillivirus (DMV) epizootics of 1990–1992 and 2006–2008, which affected Mediterranean striped dolphins (*Stenella coeruleoalba*). Between March and April 2011, the number of strandings on the Valencian Community coast (E Spain) increased.

**Case presentation:**

Necropsy and sample collection were performed in all stranded animals, with good state of conservation. Subsequently, histopathology, immunohistochemistry, conventional reverse transcription polymerase chain reaction (RT-PCR) and Universal Probe Library (UPL) RT-PCR assays were performed to identify Morbillivirus. Gross and microscopic findings compatible with CeMV were found in the majority of analyzed animals. Immunopositivity in the brain and UPL RT-PCR positivity in seven of the nine analyzed animals in at least two tissues confirmed CeMV systemic infection. Phylogenetic analysis, based on sequencing part of the phosphoprotein gene, showed that this isolate is a closely related dolphin morbillivirus (DMV) to that responsible for the 2006–2008 epizootics.

**Conclusion:**

The combination of gross and histopathologic findings compatible with DMV with immunopositivity and molecular detection of DMV suggests that this DMV strain could cause this die-off event.

## Background

Over the last 20 years, epizootics caused by *Cetacean morbillivirus* (CeMV) (genus *Morbillivirus*, family *Paramyxoviridae*) have occurred among cetacean populations worldwide and have caused mass mortality [1]. Three main CeMV groups have been described: dolphin morbillivirus (DMV) [2], porpoise morbillivirus (PMV) [3] and pilot whale morbillivirus (PWMV) [4,5].

The spread of DMV infection in striped dolphins (*Stenella coeruleoalba*) in the Mediterranean Sea caused around 1000 deaths in 1990–1992 [6]. This outbreak started in 1990 in the Gulf of Valencia, in the Spanish Mediterranean Sea [7], and propagated along European Mediterranean coasts over the following months [2,8]. Then in 2007, a new DMV outbreak occurred off the Spanish Mediterranean coast. It affected approximately 100 striped dolphins [9] and up to 60 long-finned pilot whales (*Globicephala melas*) [10], and subsequently spread to the French Mediterranean coast [11].

In the last two decades, the annual mean mortality rate of dolphins stranded off the Mediterranean coast of Valencia has been 28.4 animals per year. However this rate lowers to 18.3 animals per year if the 1990 and 2007 outbreak years are excluded [12].

In the present study, 37 dolphins are reported as stranded dolphins in 2 months, which represents more than the annual mean in that region. An evaluation of *Morbillivirus* infection revealed the overwhelming positivity of the stranded animals, suggesting that DMV could be responsible for this increase in strandings, which might be the third DMV epizootic in the Mediterranean Sea.

## Case presentation

Thirty-seven dolphins stranded along the Valencian Mediterranean coast between March and April 2011: 26 striped dolphins (*S. coeruleoalba*), three bottlenose dolphins (*Tursiops truncatus*) and eight dolphins of undetermined species (poor level of conservation hampered species identification).

Necropsies were performed according to standard protocols of the European Cetacean Society [13]. Stranded dolphins were recovered from the Valencia Mediterranean coast of Spain (39°N, 0°W) by the Marine Mammal Stranding Network of the *Conselleria de Infraestructuras, Territorio y Medio Ambiente* of Valencia. A detailed post-mortem examination could be carried out on 11 animals (nine striped dolphins and two bottlenose dolphins) since other animals were poorly preserved.

Fresh tissue samples (brain, lung, kidney, liver, lymph node, tonsil, thymus, spleen and skin) were fixed in 10% neutral buffered formalin for histopathology, refrigerated for microbiology, and tissue samples were frozen for molecular diagnosis. Immunohistochemical staining with a Canine Distemper Virus monoclonal antibody specific for nucleoprotein, IgG2B isotype (CDV-NP. VMRD®, Inc.), was carried out on selected samples of brain, lung, kidney, urinary bladder, stomach and intestine. Frozen tissues were homogenized using a Bullet BlenderTM (Next Advance, Inc., Averill Park, NY), and total nucleic acid was extracted using the NucleoSpin RNA II Kit (Macherey-Nagel) for RNA extraction and the High Pure PCR Template Preparation Kit for DNA extraction, following the manufacturers’ instructions in both kits. For the molecular CeMV diagnosis, real-time RT-PCR assays, based on the Universal Probe Library (UPL) platform, that target, a sequence within the fusion protein gene, was carried out [14].

CeMV infection was confirmed by sequencing the real-time RT-PCR products. For the phylogenetic analysis, in addition to the fusion protein gene, the DMV phosphoprotein (P) and nucleoprotein (N1 and N2) genes were amplified by conventional RT-PCRs assays according to published protocols [4,15] in some positive sample. A BLAST analysis was used to compare the obtained phosphoprotein and nucleoprotein sequences with all the CeMV sequences available in GenBank. A phylogenetic analysis was performed using the MEGA 4.0 software [16]. P-distance matrices were calculated, and tree topology was inferred by the neighbor-joining maximum composite likelihood method to test the reliability of the topology by bootstrapping 1000 replicates generated with a random seed.

*Brucella* spp. and *Toxoplasma gondii* (*T. gondii*) diagnoses were carried out in the animals whose non suppurative encephalitis was observed in the histopathological analysis. The molecular identification of *Brucella* spp. was performed in the brains of suspected animals by TaqMan Real time PCR, targeting the insertion sequence IS711 of *Brucella* spp. [17]. Additionally, *T. gondii* DNA was detected by nested PCR, in which the target formed part of the sequence of repetitive gene B1 (194 bp, 97 bp) using the method described by Montoya et al. [18].

**Figure 1:**
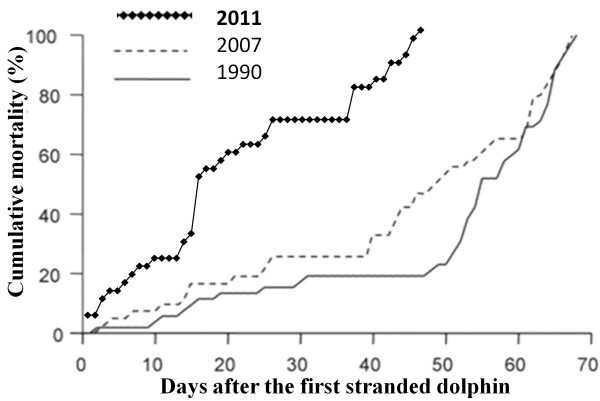
**Comparison of cumulative percentages of dolphins stranded in 1990, 2007 and 2011 epizootics.** The 2011 curve represents % between March and April 2011. 1990 and 2007 curves shows % between July and August 1990 and 2007. In 2011 curve, day 0 corresponds to 12^th^ of March 2011. Data from 1190 and 2007 are extracted from Raga et al. 2008.

Thirty-seven dolphins were stranded on the Mediterranean coast of Valencia (Spain) in mid-2011. The epizootic started at the beginning of March 2011 with a low stranding rate, but gradually increased during this month (Figure 1).

A widespread poor body condition was observed in the necropsies. Mainly gross findings were localized in the central nervous, respiratory, lymphoid and digestive systems (Table 1).

**Table 1:** **Necropsy, histopathology and morbillivirus detection results in the *Stenella coeruleoalba *individuals stranded in the 2011 epizootic**

**Stranding date (2011)**	**Age (length, cm)**	**Sex**	**Main gross lesions**	**Main histological lesions**	**Tissues tested for CeMV (positive samples underlined by UPL RT-PCR)**
**March 12**	New born (108.7)	Male	Poor body condition, severe pneumonia	Focal broncointersticial pneumonia with few giant cells, necrotic areas in reticular and perivascular cells of lymph nodes	Brain, Lung, Pulmonary lymph node, Kidney
**March 13**	New born (112.3)	Male	Numerous injuries, severe enteritis in large intestine	Multifocal lymphocytic bronchointerstitial pneumonia, bronchoalveolar hemorrhages, eosinophilic lymphadenitis, nephritis	Brain, Thymus, Lung, Pulmonary lymph node, Kidney
**March 15**	Sub adult (170)	Female	Suboptimal body condition, gas bubbles in meninges vessels, severe pneumonia, mild gastritis	Severe non-suppurative meningoencephalitis, intranuclear inclusion bodies mainly in astrocytes	Brain, Lung, Prescapular and Pulmonary lymph nodes, Kidney
**March 16**	Adult (206)	Female	Poor body condition, severe pneumonia, enteritis in large intestine with numerous parasitic granulomas	Verminous bronchopneumonia, chronic nephritis	Brain*, Lung, Pulmonary lymph node, Kidney, Liver
**March 23**	Subadult (209)	Female	Cachexia, injuries, severe brain congestion, internal hemorrhage, atelectasis, large pulmonary bullae	Verminous bronchopneumonia, pleuritiswith bacterial colonies, systemic intravascular bacterial embolism	Brain, Lung, Pulmonary lymph node, Kidney
**March 25**	Subadult (170)	Female	Pneumonia in cranial left lung, enlarged and congestive prescapular and pulmonary lymph nodes	Severe non-suppurative meningoencephalitis	Brain, Lung, Pulmonary lymph node, Kidney, Tongue ulcer
**March 26**	Subadult (176)	Female	Poor body condition, maxilla and mandible fracture, brain congestion, numerous injuries, pneumonia, atelectasis, enlarged and hemorrhagic pulmonary and abdominal lymph nodes, hepatic congestion	Mild non-suppurative meningoencephalitis	Brain, Lung, Pulmonary lymph node, Kidney
**March 29**	New born (123)	Male	Suboptimal body condition, temporal fracture, tonsillitis, tongue ulcers, pulmonary fibrosis, severe pneumonia in right lung, enlarged and congestive pulmonary lymph nodes, severe enteritis in large intestine	Severe brain hemorrhages	Brain, Lung, Pulmonary lymph node
**April 5**	New born (109)	Female	Poor body condition, pneumonia, atelectasis, enlarged and congestive pulmonary lymph nodes, tonsillitis, enteritis in both small and large intestines	Not evaluated	Brain, Lung, Pulmonary lymph node

*positive to phosphoprotein gene conventional RT-PCR.

**Figure 2:**
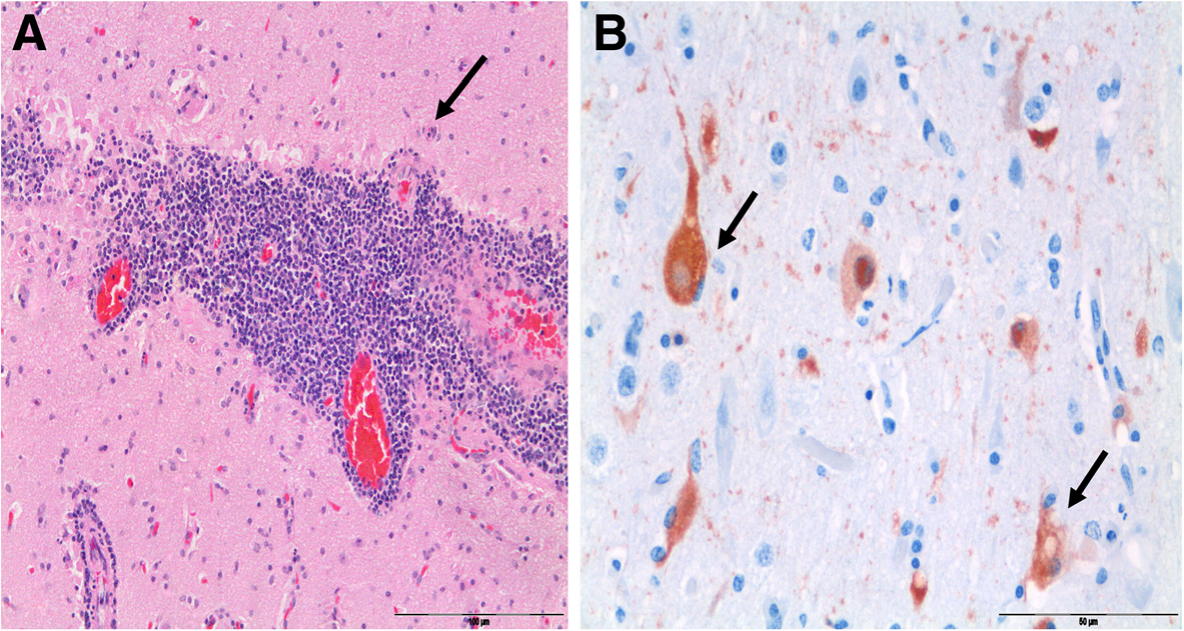
**Brain dolphin with morbillivirus infection.** (**A**) Severe non-suppurative encephalitis with acidophilic intranuclear viral inclusions (arrows) (H&E) Original amplification ×20. (**B**) Positive immunoperoxidase staining of morbilliviral antigen in glial cells and astrocytes of the brain (arrows). Avidin-biotin-peroxidase with Harris hematoxylin counterstain. Original magnification × 40.

The histopathological analysis showed severe non suppurative meningoencephalitis with numerous intranuclear inclusion bodies in three individuals (Figure 2B). Perivascular cuffing with many layers of mononuclear cells were found to especially affect inflammatory meningeal areas, vessels of cortical gray matter and, to a lesser extent, in the white matter areas. Positive immunostaining revealed the Morbillivirus antigen in glial cells and astrocytes of the brain in one of the three individuals (Figure 2B). Focal bronchointerstitial pneumonia with few giant cells was observed in some animals. However in lymphnodes, the necrotic areas in reticular and perivascular cells were found. No immunopositivity was revealed in either the lungs or lymph nodes from any animal.

According to the molecular diagnosis performed by UPL RT-PCR assays, seven of the eleven analyzed dolphins were positive in DMV/PMV UPL PCR, which represents 63.6% of positivity to CeMV. They were all striped dolphins. After considering that the most affected specie in the last Mediterranean DMV epizootics was striped dolphin, the percentage was calculated in relation to all the analyzed striped dolphins, which changed to 78% of positivity (seven positive striped dolphins as compared to nine analyzed striped dolphins) (Table 1). Furthermore, the systemic form of the disease was found in five of these seven animals, which contained at least two positive tissues. However, only one of the seven positive animals analyzed by UPL RT-PCR was positive for phosphoprotein by conventional PCR, and this positivity was restricted to brain.

**Figure 3:**
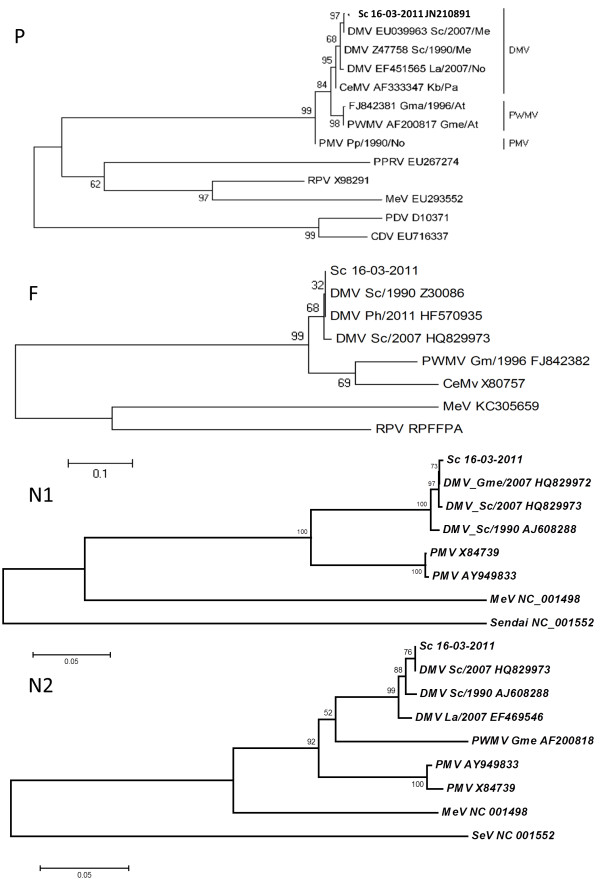
**Neighbor-joining phylogram of morbillivirus phosphoprotein (P), fusion (F) and nucleoprotein (N1 and N2) genes sequences.** The name of each sequence is composed of the virus name (DMV, dolphin morbillivirus; CeMV, Cetacean morbillivirus; PMV, porpoise morbillivirus; PWMV, pilot whale morbillivirus; PPRV, Peste-des-Petits-Ruminants virus; RPV; Rinderpest virus; CDV, Canine Distemper virus; PDV, Phocine Distemper virus; MeV, Measles virus), GenBank accession number, cetacean species infected (Sc, *Stenella coeruleoalba*; Gme, *Globicephala melas*; La, *Lagenorhynchus albirostris*; Kb, *Kogia breviceps*; Tt, *Tursiops truncatus*; Pp, *Phocoena phocoena*; Gma, *Globicephala macrorhynchus*), the year and the geographical area of the stranding (At, Atlantic Ocean; Me, Mediterranean Sea; No, North Sea; Pa, Pacific Ocean).

Sequencing the amplicons from the fusion protein gene (F) [14], the phosphoprotein gene (P) [15] and the two fragments of the nucleoprotein gene, named N1 [5] and N2 [19], confirmed infection by DMV. The phosphoprotein gene sequence obtained from the brain of one striped dolphin (GenBank accession number JN210891) showed a p-distance of 0.003 with the 2007 Spanish strain (GenBank EU039963) [10] and of 0.015 with the 1990 Spanish strain (GenBank AJ608288). Thus, the phosphoprotein gene sequences for the 2007 and 2011 Spanish strains were 98.5% identical (Figure 3). In addition, the fusion protein sequences showed 100% identity with the 1990 Spanish strain (GenBank AJ608288) and the 2007 Spanish strain (GenBank accession number HQ829972) [20] (Figure 3). Complementarily, the N2 fragment of 495 bp [19] and the N1 fragment of 181 bp of the nucleoprotein gene [5] were compared with other DMV nucleoprotein gene sequences, which confirmed that the 2011 DMV strain evolved from the 1990 DMV strain and the 2007 DMV strain (Figure 3). The N1 sequence was 100% identical to the others observed in the Mediterranean Sea from 2007 to 2012 [20,21], whereas the N2 fragment showed a similarity of 99.8% with the DMV sequence obtained from the *Globicephala melas* mass stranding of 2007 [20].

The microbiological investigations found no pathologically significant microorganisms. As regards *Brucella* spp. and *T. gondii* PCRs, negative results were identified in the three animals with non suppurative encephalitis.

The 37 strandings in just 2 months might represent an unusual mortality event if we consider the annual number of stranded dolphins has not been exceeded since 1990, except in 1990 and 2007, on Valencian Mediterranean coasts [12]. DMV was considered the causative agent of this increase in strandings in 1990 and 2007 [7,9]. In addition, this is not the first description of DMV in the Mediterranean Sea in 2011. In Italy, DMV has been reported in striped dolphins, bottlenose dolphins and fin whales on almost the same dates as this report [21-24]. Accordingly, Morbillivirus infection finding might be related with the rise in strandings in 2011.

DMV detection by the UPL RT-PCR assay in 78% of the analyzed striped dolphins highlights the important role that DMV plays in this unusual episode of mass strandings. At the same time, the recognition of DMV in at least two tissues in five animals may indicate the general spread of this virus, most likely by the circulatory system. Failure to detect CeMV in the central nervous system (CNS) in all the positive animals can be explained by the fact that distribution of CeMV brain infection is not homogeneous [25] and that the CNS samples in this study were not collected uniformly; thus it was impossible to determine from which brain region each sample had been taken. In addition, *Toxoplasma gondii* and *Brucella* spp. have been related with encephalitis in stranded dolphins [26,27], and even together with CeMV [22]. However in our study, any of the three cases with non suppurative encephalitis can be related with these pathogens.

A comparison made of the striped dolphin outbreaks in 1990, 2007, and 2011 suggests a change in DMV epidemiology in the Western Mediterranean Sea. In 2011, mortality was even lower, lesions were less severe, and mostly younger animals were affected. Since enzootic infections in wildlife are characterized by milder lesions and lighter pathogen loads than epizootics [28], it is possible that DMV epidemiology in striped dolphins in the Western Mediterranean is changing from epizootic to enzootic infection, as suggested by others [20,25]. Systematic serological surveys are urgently required to address this question.

## Conclusions

In conclusion, the presence of DMV-compatible lesions, the antigen detection in one of the animals and the molecular detection of DMV genomic sequences all suggest that DMV is associated with this unusual mass mortality episode in striped dolphins in the Western Mediterranean Sea. Further research to define how the virus circulates and causes epidemics in the Mediterranean Sea, and why only striped dolphins were affected in 2011, is warranted.

## Abbreviations

CeMV: Cetacean morbillivirus; DMV: Dolphin morbillivirus; RT-PCR: Reverse transcription polymerase chain reaction; UPL: Universal probe library; PMV: Porpoise morbillivirus; PWMV: Pilot whale morbillivirus; S. coeruleoalba: Stenella coeruleoalba; CNS: Central nervous system.

## Competing interests

The authors declare that they have no competing interests.

## Authors’ contributions

Necropsies were performed by JLC, CRG and MM; microscopic examination and immunhistochemistry were carried out by ES and MA; the viral study and the phylogenetic study were analyzed by CRG, MM, NEB, and FE; the manuscript was prepared and critically discussed by CRG, MM, FE, NEB and JMSV, with contributions by all the remaining authors. All the authors read and approved the final manuscript.
